# Evaluating the Impact of Emergency Department Length of Stay in a Military Training Hospital Following the Implementation of a Standardized Paging System

**DOI:** 10.7759/cureus.62102

**Published:** 2024-06-10

**Authors:** Steven Siemieniak, Skylar Dehaan, Aaron Matlock

**Affiliations:** 1 Emergency Medicine, Brooke Army Medical Center, Fort Sam Houston, USA; 2 Emergency Medicine, San Antonio Uniformed Services Health Education Consortium, Brooke Army Medical Center, Fort Sam Houston, USA

**Keywords:** time of admission, computer-based consultation, consultation time, consult, ed length of stay

## Abstract

Emergency department (ED) lengths of stay (LOS) may be unnecessarily extended by inefficient consulting processes. Delays in initiating consultations, returning calls, consultant evaluation of patients, and communication of recommendations can contribute to potentially avoidable increases in LOS. Prolonged ED LOS has been shown to increase patient morbidity and mortality and to decrease patient satisfaction. We created a standardized procedure for ED-initiated consultations, with the goal of reducing the time to initial consultant callback, time to admission, and total ED LOS. Following our intervention, time to consultant callback was decreased; however, there was no reduction in total ED LOS for admitted patients.

## Introduction

Emergency departments (EDs) are often challenged with managing long wait times and prolonged lengths of stay (LOS), and with ED volumes having returned to pre-COVID-19 pandemic levels, overcrowding is likely to increase [1]. ED LOS may be further compounded by system inefficiencies and delays related to a specialist or admitting service consultation, particularly if required for definitive determination of patient disposition.

Currently, many institutions, including academic and community facilities in the United States, utilize paging services to initiate ED consultations. These services include but are not limited to traditional pages to pagers, encrypted messages in the electronic health record (EHR) sent directly to the consultant, or callback numbers sent to consultants' personal cell phones. Delays in communication may occur related to pager malfunction, network downtime, or incorrectly listed or dialed pager numbers. On-call consultants may be busy with more critical tasks at the time a page is received and be unable to return the call promptly. Similarly, ED staff may not be present to receive the return phone call while tending to other patients in the department. Conceivably, such factors may be additive in their effect on throughput efficiency and LOS. Brick et al. demonstrated this cumulative effect in a tertiary ED, in which specialty consultation accounted for 33% of LOS for admitted patients and 54% of LOS for discharged patients [2]. Our military academic institution in the United States utilizes a traditional paging system, in which our medical support assistant (MSA) pages the consultant with our emergency department callback number in order to initiate a consultation. As this is an academic institution, both resident and attending physicians practice in this facility.

LOS can be impacted by additional factors, such as hospital bed availability, coordination of nursing assignments, and timing of admission orders placed by consultants. We specifically focused on our quality improvement project, with the intention of demonstrating that decreasing consultation time could lead to reductions in ED LOS. 

Shen et al. demonstrated that automation of certain ED processes, including consultation, could decrease LOS despite an increase in ED patient volume [3]. Furthermore, they demonstrated that the lack of a standardized consultation process led to a variation of up to two hours in patient disposition times, when corrected for patient load [3]. Systematic reviews have identified standardization of paging workflows as effective strategies to decrease ED LOS [4,5] with median reductions ranging from 36 to 52 minutes in individual studies [6,7].

In January 2022, our facility implemented MHS Genesis, the new EHR of the Defense Health Agency. This EHR allows for improved tracking of ED throughput metrics and provides opportunities to increase departmental efficiency and decrease ED LOS for admitted patients. Using tools available in MHS Genesis, we sought to implement a standardized paging process in hopes of improving the efficiency of our consulting process and decreasing one of the potential increases in LOS for patients in our department.

## Materials and methods

We defined the steps of our intervention in a written standard operating procedure (SOP) (Appendix). The SOP was implemented in order to define and set timeliness standards for contact and communication with consultants for patients in the ED. Following the new SOP, when a consult is needed, a provider places an order in the EHR to initiate the consult via paging service.

If the initial page is not returned promptly, the MSA follows a predetermined timeline for repeat pages, as displayed in Table 1, and further described in the SOP (Appendix). The MSA is empowered to take action independently, thus not requiring the ED provider to prompt or request successive pages. MSAs are civilian contractors who are specifically trained to operate our consultant SOP, in addition to coordinating inter-facility transfers and ensuring the successful uploading of supplemental medical charts and documents. If MSAs are not available, the responsibility to order the consultant order in MHS Genesis, to document the time the page was placed, and to send the initial consultant page, falls on the emergency medicine provider, either the attending or resident physician.

**Table 1:** Standard Operating Procedure Consultant Callback Protocol

Clock	Step
0 minutes	1st Page: Resident/Fellow on call
15 minutes	2nd Page: Resident/Fellow on call
20 minutes	3rd Page: Attending if listed; if not, overhead page service
30 minutes	Overhead page the consulting service

Prior to the implementation of MHS Genesis, our department could track total ED length of stay using our legacy EHR systems, but we were unable to accurately stratify pertinent metrics related to paging and consult timing. Consequently, a pre-intervention comparison could only be made for total ED LOS, when comparing MHS Genesis data to that obtained from legacy systems. 

MHS Genesis allowed for greater granularity in tracking consult process efficiency, as our workflows in MHS Genesis dictate that an order be placed at three key time points: the time a consult is placed, the time the ED provider speaks with the consultant (a “request for admit” order), and the admit order placed by the consultant. By comparing the time intervals between these respective orders before and after the implementation of our SOP, we were able to track and compare differences at each stage, in addition to ED LOS.

ED throughput data were analyzed and compared across several time periods, pre- and post intervention. For historical comparison, the average ED LOS data for admitted patients were compiled for the year and month prior to our intervention, using data from our legacy EHR and MHS Genesis. In the week prior to the implementation of our paging SOP (May 9-15, 2022), ED providers were instructed to order consults using EHR order entry, but other aspects of the SOP were not yet implemented. On May 16, our SOP was initiated. ED LOS and consultant-specific time interval data were collected from May 9, 2022, through July 31, 2022.

**Figure 1:**
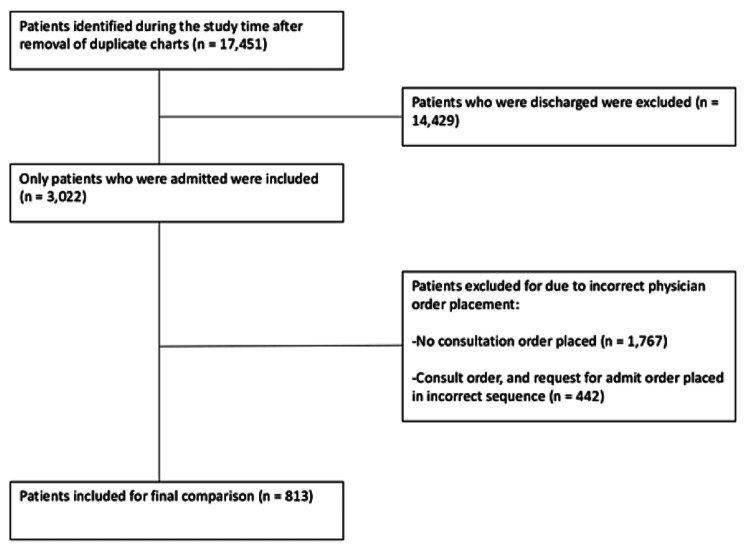
Final Inclusion Criteria for Data Analysis

During the study period, 17,451 patients were seen in our ED. Specifically, 15,980 patients were seen after the implementation of the system, and 1,471 were seen during the week used as the comparison group. Given the objectives of this comparison, data points were pulled for admitted patients only. This left 2,778 patients admitted over the study time frame, compared to 244 in the comparison group. Finally, only charts with proper consultation and requests for admission orders could be included to allow for proper comparison, including charts where orders were placed out of sequence. Therefore, 760 patients and 53 patients in the comparison group met the final inclusion criteria. These data are visualized in Figure 1. 

After entering the data into Excel for comparison and analysis, duplicate orders were first removed using conditional formatting to identify repeat patient identifiers. Once the duplicate orders were removed, patients were organized by patient identifier number (PIN).

We used tools intrinsic to MHS Genesis to specifically analyze patient arrival time: “consult to medicine” or “consult to surgery” orders, “request for admit” orders, and patient departure time. Each order was matched with the PIN used for that visit. A comparison of the differences in these order times was used to determine the amount of time required for each step in the admission process.

The difference between patient arrival and departure times was used to represent the total ED LOS. The difference between patient arrival time and the order for consultation functioned as a surrogate for the time required to be seen by an ED provider and the admission decision to be made (time to disposition). The interval between the consultation order and the patient's departure time represented the time required for the consultant to see the patient, place admission orders, and for the patient to leave the department. Averages of these time intervals were compared for the pre- and post-intervention time periods. Patient arrival time did not discriminate between total time in the waiting room versus patients' who were immediately roomed based on acuity.

The launch of our new paging system necessitated the dutiful placement of consult orders by the providers and the disciplined entry of subsequent orders following consultations. The system's complexity introduced multiple points where data could potentially be misplaced. Before our evaluation, it was common practice for ED providers to determine a patient's need for admission and issue an admission order immediately upon ED presentation. We emphasized the critical need to delay the admission order until after consulting with a specialist, aiming to capture more accurate data on response times. Recognizing the risk of data loss in this approach, we rigorously monitored the sequence of consultation and admission orders. To maintain the integrity of our data, we excluded any records where the admission order preceded the consultation order, thus eliminating the possibility of distorted results. Further, we did not include patient encounters in our study that did not have reported arrival and departure times, consultation initiation and completion, consultation to bedside time, or time from admission from consultation evaluation.

Statistical analysis was performed with the assistance of our hospital statistician, and the outcomes were compared using the Wilcoxon-Mann-Whitney two-sample rank sum test. The study was granted exemption by our institutional review board as a quality improvement study.

## Results

Following the implementation of our paging protocol, there was a significantly improved time to consultation callback after page placement. Prior to implementation, an average time of 55 minutes and 23 seconds elapsed prior to callback. Following the initiation of our SOP, the time-to-return calls were decreased to an average time of 20 minutes and 25 seconds in the post-intervention period. This demonstrated an average of 34-minute and 57-second improvement prior to the execution of the automated protocol.

Using the Wilcoxon-Mann-Whitney p values, there was a statistically significant improvement in LOS from May to June 1 (p value < 0.001), as well as from May to July (p value < 0.001). More specifically, from May 9 to May 23, there was an initial statistically significant improvement in LOS (p value = 0.007), and from May 16 to 23 (when the protocol was initiated), there was an even more significant improvement (p value < 0.001). Chi-square approximation demonstrated a probability simply to chance of less than 0.001.

Furthermore, there was a statistically significant improvement in time to admission order placement from May to June 1 (p value < 0.001), as well as from May to July (p value < 0.001). More specifically, from May 9 to May 23, there was an initial statistically significant improvement in time to admission order placement (p value = 0.0015). Chi-square approximation demonstrated a probability simply to chance of 0.0028.

Looking at the time from consultant callback to the placing of admission orders, there was a statistically significant improvement in time to inpatient admission order placement from May to July (p value < 0.001). More specifically, from May 16 to May 23, there was a statistically significant improvement in time to admission order placement to admission orders (p value = 0.0021). Chi-square approximation demonstrated a probability simply to chance of 0.001. The data did not demonstrate a statistically significant improvement in time from admission order placement to admission to the hospital.

Despite the statistically significant decrease in time to returned consultant calls, other time intervals did not similarly improve. The average total emergency department LOS increased by a range of 10-20%, with one outlier week from May 23 to 30 that demonstrated nearly a 45-minute decrease from other recorded times. Similarly, the average time for consultant evaluation of the patient also increased during protocol implementation, with the longest being approximately 45 minutes. Finally, time from returned page to admission also fluctuated, with the same outlier week from May 23 to 30.

**Figure 2:**
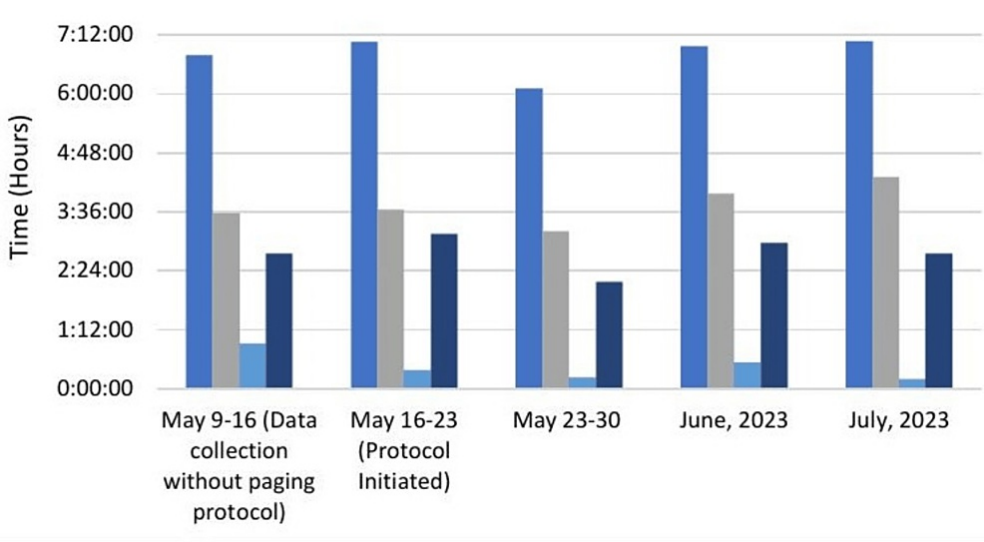
Paging Protocol Impact on Department Times Royal blue: total ED LOS; gray: ED provider evaluation time; light blue: time to consultant callback after first page; navy: consultant evaluation time to time of admission

Average times both before and after implementation of the paging system are displayed in Table 2 and Figure 2.

**Table 2:** Average Departmental Times Before and After Protocol Implementation Times displayed are in hours: minutes: seconds; average department time: from check-in to check-out; average arrival to consult: the time until we completed our workup and paged for admission; consult to returned call: the time we placed the bed request (when the consult called back); consult returned to admission: consult evaluation, orders, and patient leaving the department

Date	Average department time	Average arrival to consult	Consult to returned call	Consult returned to admission
2022 Average	6:30	/	/	/
May 9-15 (Data collection without paging protocol)	6:47:47	3:34:20	0:55:23	2:45:31
May 16-22, 2023 (Protocol Initiated)	7:03:26	3:38:52	0:23:18	3:08:49
May 23-30, 2023	6:06:52	3:12:10	0:13:28	2:10:50
June 2023	6:58:39	3:58:01	0:32:06	2:57:45
July 2023	7:04:36	4:18:48	0:12:23	2:45:48

## Discussion

The results of the standardized paging protocol significantly reduced time from consultant page to callback, ultimately leading to patients being evaluated more quickly by consultants; however, it was not associated with significant decreases in our overall ED LOS. Data analysis helped identify additional areas where ED throughput was bottlenecking. Other throughput measures that have previously been shown to reduce ED crowding, such as a provider in triage (PIT) and a fast track section, were previously implemented in our ED and remained unchanged throughout the duration of the study period [4]. Our paging system demonstrated that a low-cost intervention, such as placing consultation orders, can lead to reduced time until consultation evaluation. Previous literature has shown that consultant decision time has a significant impact on ED LOS [2]. However, despite significantly improving time to consultant page back, time to consultant evaluation, and time to order placement for admission, the overall ED LOS did not improve appreciably. This was an unexpected result, as similar processes to initiate ED consults, such as text messages, have demonstrated decreased ED LOS [7].

We suspect that this is attributable to other unmeasured variables, including additional bottlenecks downstream from the ED, such as bed availability, coordination of nursing assignments, and timing of admission orders placed by consultants. Additionally, providers in our department are often called upon to care for trauma patients, and the unpredictable arrival of such patients can interfere with the timely dispositions of non-trauma patients. We did not evaluate disposition times in association with trauma volume, nor did we stratify data by time of day. ED LOS may be longer at night or on days with heavy trauma volume, and these factors may have changed the impact of our intervention. Finally, the comparison to last year's LOS was for all patients, not just for admitted patients that were measured in this study.

We believe that the significant reduction in time to consultation and time to admission order following the implementation of our paging system was also likely impacted by increased resident buy-in, as well as the timing of the intervention occurring later in the academic year, when residents are more comfortable in their respective roles, both in the ED and in inpatient units. As time progressed through the post-intervention period, users in our hospital system were becoming more familiar with the EHR, and this may account for the changes we observed. However, in early July, internal medicine residents begin their training in our facility, and the introduction of new trainees may have accounted for increases in our measured time intervals for this month, though our study is not specifically powered to this end.

An important limitation of our data is that analysis was restricted to patients who were admitted to the hospital. Specialist consultation for patients who would ultimately be discharged likely adds significant time to ED department LOS compared to those who did not receive consultation. We see this as a future area of study in this realm. Finally, in considering the implementation of this or similar paging protocols, departments should be aware of the potential downsides of such a system. If repeat pages and/or overhead pages are initiated at times when the consultant clearly cannot return the call (e.g., a surgeon who just took a critical patient to surgery), or when the ED provider is unavailable to answer a return call, this could result in unnecessary stress and potentially damage relationships with consultants and ED providers. This could lead to negative downstream throughput effects such as inpatient beds not being cleared, as demonstrated in previous studies [8].

## Conclusions

With this limited dataset, we demonstrated that a standardized paging protocol significantly reduced the time from the consultant page to callback. This intervention led to patients being evaluated more quickly by consultants, but it was not associated with significant decreases in our overall ED LOS. A similar system has been shown to have positive results in other systems; therefore, more research is needed to assess if external factors may be impacting this result.

## Appendices

MEMORANDUM for the Department of Emergency Medicine, Brooke Army Medical Center, 3551 Roger Brooke Drive, JBSA Fort Sam Houston, Texas 78234-4504SUBJECT: Standard Operating Procedure (SOP) for Contacting Consultants in the Emergency Department

1. PURPOSE: Defining and setting timeliness standards for contact and communication with consultants for patients in the Emergency Department.

2. SCOPE: This SOP is intended to provide guidelines for Emergency Department providers, nurses, and Medical Support Assistants (MSAs).

3. BACKGROUND: The timeliness of specialty consultation to the Emergency Department is defined in the Medical Staff By-Laws with the following: “Departments will provide consultation support to the ED and/or Clinical Decision Unity promptly; within one hour of being consulted.” This time does not take into account the time it takes the ED provider to page the consultant (or ask the MSA to page), the page to go out, the page to be returned, and the conversation between the ED provider and consultant to occur. Additionally, ED Providers often have multiple simultaneous tasks and may be actively attending to another patient or occupied elsewhere in the department when the page is returned which causes the ED provider to miss the call and have to re-page a consulting service. The other source of delay is when the ED provider pages the consultant directly either by personal preference or when an MSA is unavailable in the Pod - if that ED provider then gets pulled away from their desk phone and misses the return call, there is no way to know and they have to re-page a consulting service. The implementation of MHS-Genesis provides a potential improvement in that consults can be ordered in the system. When a consult is ordered, it is time-stamped, the MSA can then see the consult and send the page, and an accurate recording of the timeliness of the consult can be accomplished. This system is still imperfect in that the ED provider may get pulled away when the page is returned; however, it would be an improvement over the historical system.

4. WORKFLOW, RESPONSIBILITIES, POLICIES, AND PROCEDURES:

a. Once the ED Provider determines that specialty consultation is necessary, an order is placed in MHS-Genesis for the consult (i.e. “Consult to Internal Medicine”). The MSA assigned to the Pod is responsible for acknowledging the order and placing the page to the on-call resident pager. Closed-loop communication is important between the ED Provider and the MSA to ensure that the MSA is made aware of the order for the consult in a timely manner. The callback number on the page should be to the MSA, who is more likely to be available to answer the phone. A timeline for paging consultants is listed below, and the number for the on-call residents and staff physicians can be found on Amion for most services.

b. If a return call is received and the ordering ED provider is not immediately available, the MSA would then page overhead in the department that a call is waiting. If the ED provider is unable to take the call (i.e. attending to a critical patient or otherwise), the ED provider can designate another provider to give the message. Meanwhile, the MSA can provide the consultant with the name and room number of the patient and can take the consultant’s name and number to be called back.

c. The ED Provider will:

(1) Determine the need for consult

(2) Place the order for the consult in MHS-Genesis

(3) Closed loop communication with the MSA about order

(4) Be available and alert for the return call

d. The MSA will:

(1) Be aware of the process, procedure, and how to access current pager numbers.

(2) Upon an order for a consult, execute the following steps:

Steps:

0 minutes 1st page: Resident/fellow on call

15 minutes 2nd page: Resident/fellow on call 20 minutes

20 minutes 3rd page: Attending if listed, if not overhead page service

30 minutes Overhead page the consulting service 

(3) Successive pages do not require approval from the ED provider, the MSA can take that action independently.

(4) Document attempts and outcomes.

5. The point of contact for this action is the undersigned at 210-916-7028 or by email at wesley.e.trueblood.mil@mail.mil. WESLEY E. TRUEBLOOD, MD, Lt Col, USAF Service Chief, Department of Emergency Medicine
